# Combining Abdominal Hernia Repair With Abdominoplasty—Is it Safe?

**DOI:** 10.1093/asjof/ojag069

**Published:** 2026-04-21

**Authors:** Leonardo Alaniz, Nikhil Prabhakar, Gregory R D Evans

## Abstract

**Background:**

Increasing obesity rates, GLP-1 receptor agonist adoption, and demand for body contouring procedures have created a population requiring both functional hernia repair and aesthetic abdominoplasty.

**Objectives:**

To evaluate the safety and feasibility of concurrent abdominoplasty and hernia repair using a standardized approach.

**Methods:**

We conducted a retrospective case series on patients undergoing simultaneous abdominoplasty and ventral hernia repair at a single academic center (2018-2024). Primary outcomes included 30-day surgical site occurrences (SSOs), hernia recurrence, and reoperation rates. Secondary outcomes included operative efficiency metrics and patient-reported satisfaction. Statistical analysis used chi-square and 2-sided t-tests; *P* < .05 was significant.

**Results:**

This cohort included 20 patients. Mean age was 58.2 years, 75% were female, and there was substantial comorbidity burden (mean BMI 30.8 kg/m^2^; majority overweight or obese; 60% had prior hernia repair). Midline ventral/incisional, umbilical, inguinal, and complex/recurrent hernias were represented. Mesh reinforcement was utilized in 50% of cases. At a mean follow-up of 27.3 months, SSO occurrences were observed in 3 patients (15%): 2 seromas (10%) and one case (5%) of chronic drainage that resolved with bedside care. No hematomas, mesh infections, or hernia recurrences were identified. Mean total operative time was 306 minutes; reconstructive and aesthetic components averaged 115 and 167 minutes, respectively.

**Conclusions:**

In this limited cohort of carefully selected patients, concurrent abdominoplasty with ventral hernia repair demonstrated an acceptable safety profile, with complication rates comparable to historical abdominoplasty alone benchmarks and no recurrences at intermediate follow-up. Larger prospective studies are warranted to refine patient selection and standardize best practices.

**Level of Evidence: 4 (Therapeutic):**

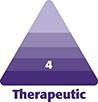

According to the most recent global survey, over 34.9 million aesthetic procedures were performed by plastic surgeons in 2023, reflecting a 3.4% annual increase. This included more than 15.8 million surgical and 19.1 million nonsurgical procedures. Aesthetic procedure volume has steadily increased over the past decade, with a 40% overall increase seen in just the last 4 years, driven by particularly sharp growth since 2021.^1^ The steady increase in aesthetic procedures in the past decade highlights the importance and demand for plastic surgery as more patients opt for surgical procedures. As life expectancy increases, patients are also more likely to pursue surgical procedures to improve their physical appearance. With the increased utilization of Glucagon-like peptide-1 receptor agonists (GLP-1), the amount of surgery for excess weight loss and lipodystrophy will increase.^2,3^

Upon ensuring patient safety and proper outcomes, concurrent procedures have been studied, attempting to increase efficiency and reduce costs on both the hospital and patient. Early combined surgical procedures, such as cesarean sections combined with hysterectomies, paved the way for modern concurrent surgeries by addressing multiple patient needs in a single procedure. Abdominoplasty is sought by patients to remove excess abdominal skin and fat, often after significant weight loss or pregnancy.^4^ For abdominoplasty procedures, the most common complications are infection, seromas and hematomas.^5,6^ However, many patients undergoing abdominoplasty also present with ventral hernias, such as incisional, umbilical, or inguinal hernias, which pose significant challenges during abdominal procedures due to risks of postoperative complications.^7^ Existing literature that follows traditional techniques for abdominoplasty and umbilical hernia repair, for example, claims that the vascular supply to the umbilicus can become compromised, as the umbilical stalk becomes dependent on the deep inferior epigastric artery blood supply after incision of the dermal plexus.^8^

Hernia repair, including a large proportion of ventral hernias, remains one of the most frequently performed general surgical procedures, with over 600,000 hernia repairs performed annually in the United States.^9^ By investigating how these surgeries can be performed concurrently, patient outcome and surgical efficiency can both be improved. This overlap in patient populations underscores the importance of exploring combined surgical interventions. Performing abdominoplasty concurrently with hernia repair offers a unique opportunity to address these interrelated conditions while potentially minimizing complications and optimizing surgical efficiency.

Although concurrent procedures seem to improve provider efficiency and patient recovery, further research is needed to investigate the effects on patient outcome and underlying safety. In a large study utilizing data from the American College of Surgeons' National Surgical Quality Improvement Program (NSQIP), researchers evaluated a broad range of concurrent surgical procedures, including general surgery, orthopedic, and urologic operations. After adjusting for patient and procedural factors, the study found no significant associations between performing concurrent procedures and increased risks of serious morbidity, unplanned reoperation, or readmission.^10^ These findings suggest that, when appropriately selected and managed, concurrent surgeries may offer clinical utility without substantially increasing postoperative risk. Our study aimed to evaluate the efficacy and safety of combining abdominoplasty and hernia repair procedures. A retrospective review of patient outcomes was performed to determine whether this approach could reduce hernia recurrence and improve overall surgical outcomes while minimizing complications.

## METHODS

Between 2018 and 2024, we retrospectively identified consecutive adult patients who underwent combined abdominoplasty and ventral hernia repair at a single academic center. This retrospective study was reviewed by the University of California, Irvine Institutional Review Board (Protocol #3351) and determined to be exempt under 45 CFR 46.104(d)(4). The IRB granted a waiver of informed consent and a waiver of HIPAA authorization for use of de-identified data. After reviewing patient electronic medical charts, patients' comorbidities and descriptive statistics were studied and analyzed for trends. Comorbidities recorded for patients included obesity, diabetes mellitus, hypertension, hyperlipidemia, smoking history, prior abdominal surgeries, connective tissue and immune disorders. High risk patients that were pregnant and/or had major comorbidities relating to cardiovascular (eg, uncontrolled hypertension, coronary artery disease, congestive heart failure), hepatic (eg, chronic hepatitis with impaired liver function) or renal function (eg, chronic kidney disease stage III or higher) were excluded from the study.

Upon analysis of patient charts and operative notes, time points of each combined surgery were taken to split concurrent procedures into reconstructive and aesthetic portions. A plastic surgeon was responsible for opening the abdomen, which was inclusive of exposure for the reconstructive process. Afterwards, a general surgeon performed the hernia repair, and the respective times were recorded appropriately. The plastic surgeon then closed the abdomen and added plication, which was considered the aesthetic portion of the procedure. These times were added appropriately to calculate portions of operative time by each surgeon respectively. Primary outcomes included 30-day surgical site occurrences (SSO) or postoperative complications. Hernia recurrence was assessed clinically and/or radiographically at last follow up. Postoperative complications were defined as any adverse event occurring within 30 days of the concurrent procedure. These were classified as minor (seroma requiring aspiration, superficial infection, small wound dehiscence, delayed healing) or major (hematoma requiring evacuation, flap necrosis, deep infection requiring intravenous antibiotics or reoperation, venous thromboembolism, or hospital readmission). Incomplete chart information was excluded from statistical analysis, which included specific time breakdowns of aesthetic vs reconstructive portions of the concurrent surgery for 3 patients. Chi-square and 2-tailed t-tests were performed for statistical analysis, with significance achieved when *P* < .05.

Exposure of the abdominal hernia depended on location and occurred through the routine exposure for an abdominoplasty through a low Pfannenstiel incision consistent with those done for abdominoplasty alone.^11^ The abdominal skin was elevated up to the costal margins and the xiphoid process. The infra-umbilical skin was divided at midline which allowed easier elevation up to the superior identification points. Care had to be taken to not injure the intestines which may have been protruding through the anterior rectus fascial wall with the abdominal hernia. When possible, the umbilicus was maintained. In several patients however, the extent of the hernia required removal of the umbilicus and was decided intraoperatively by the multidisciplinary surgery teams. When the native umbilicus was excised, a neoumbilicoplasty was performed using a transcutaneous purse-string technique with dermal fixation to the rectus fascia. However, immediate neoumbilicoplasty was not performed, as recommended by the general surgeons, to prioritize definitive hernia repair and resection of redundant abdominal skin without introducing additional procedural complexity or morbidity. In these cases, umbilical reconstruction was deferred as an elective procedure that patients could opt for if they desired.

**Figure 1.**
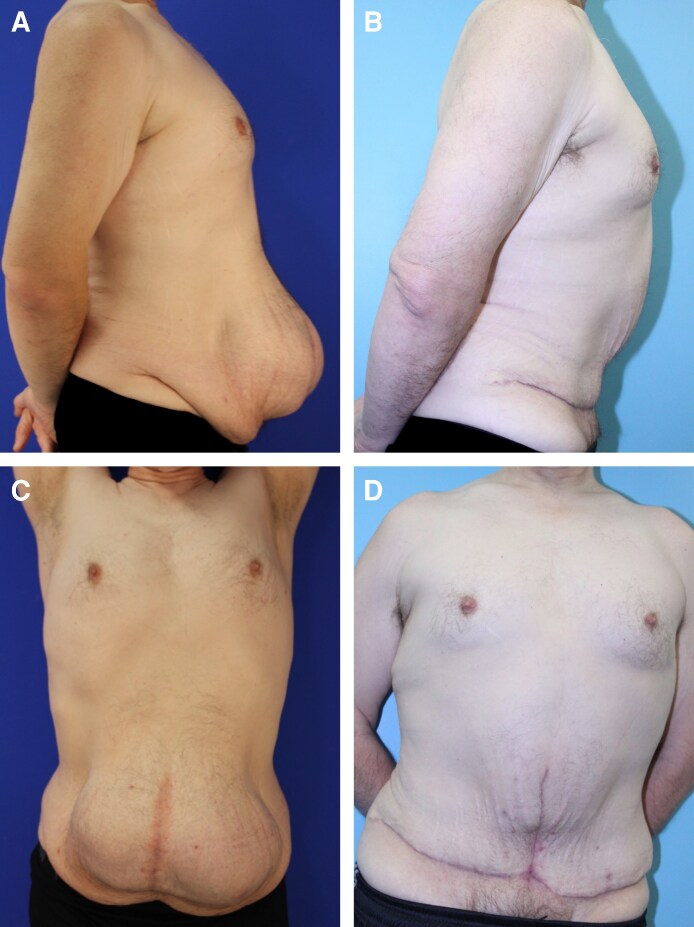
Fifty-three-year-old man with abdominal lipodystrophy and ventral hernia who underwent ventral hernia repair with mesh and abdominoplasty, 6 months postop. (A) Preoperative photo, side profile; (B) postoperative photo, side profile; (C) preoperative photo, front profile; (D) postoperative photo, front profile.

**Figure 2.**
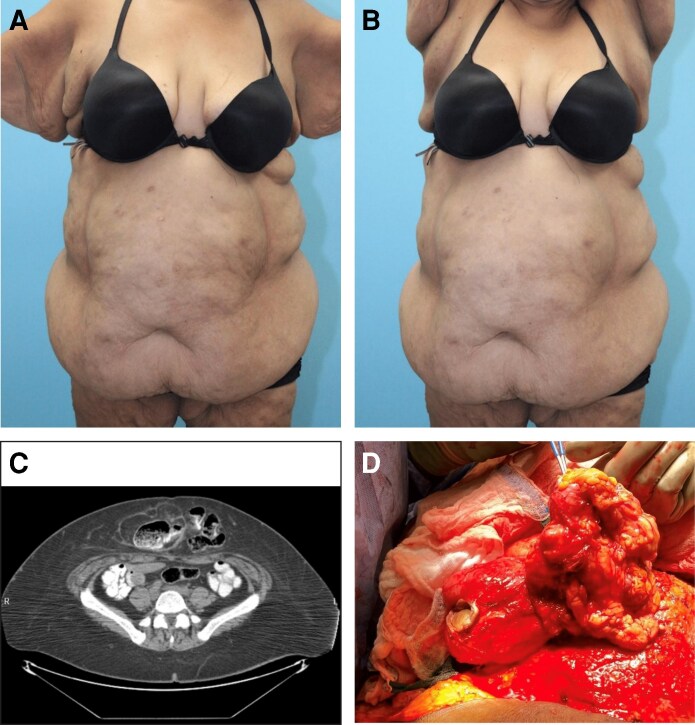
Forty-five-year-old woman with abdominal lipodystrophy and umbilical hernia, 6 months postop. (A) Preoperative photo, anatomic position. (B) Preoperative photo, arms out position. (C) Axial computed tomography showing umbilical hernia. (D) Intraoperative photo.

This outpatient procedure was performed by general surgery, who repaired the abdominal hernia by opening and retracting the peritoneal and hernia sac and repositioning any protruding intestines. Finally, the surgical team closed the abdominal hernia sac with nonabsorbable suture with or without mesh (Figure 1). The decision for mesh reinforcement was based on defect size: primary suture repair was performed for defects <3 cm without tension, while mesh reinforcement was used for defects ≥3 cm, recurrent hernias, or multiple defects. Frequently the abdominal incisional hernia was in the midline allowing traditional plication over the abdominal incisional hernia closure. This was usually done with a nonabsorbing interrupted figure-of-eight suture, as well as a running barbed suture overlaying the figure-of-eight closure (Figure 2). Any additional lateral plication was performed with interrupted nonabsorbable sutures.

Standard closure of the abdomen then proceeded with excision of excess tissue, placement of drains, injection of pain medication through abdominal wall blocks and re-approximation of the umbilicus if it remained. Postoperative care included stool softeners, adequate closed-suction drains, abdominal binder after 2 to 3 days, ambulation and DVT prophylaxis, if necessary. All patients received mechanical prophylaxis with sequential compression devices (SCDs) intraoperatively and postoperatively until full ambulation. Risk stratification used the Caprini Risk Assessment Model. Patients with Caprini scores ≥5 received chemoprophylaxis with low-molecular-weight heparin unless contraindicated.^12^ Chemoprophylaxis was initiated postoperatively once hemostasis was assured and continued during the inpatient observation or for a short outpatient course in selected high-risk patients

Patients were discharged the same day when pain was controlled, oral intake tolerated, and early ambulation achieved. If further concerns for these functions were not met, the patient was admitted for 23 hour observation. Postoperative counseling was provided by the operating plastic surgeon to ensure adherence to wound care and activity restrictions. Patients were instructed on wound monitoring, drain management, and medication use to promote healing, optimize pain control, and facilitate early recognition of complications. Early ambulation and continuous use of compression garments were recommended to support abdominal wall repair and reduce seroma formation. To protect the integrity of fascial closures, patients were advised to avoid straining, twisting, or lifting objects heavier than 5-10 pounds (2-5 kg) for the first 4 to 6 weeks.

## RESULTS

The study included a total of 20 adult patients. Mean patient age at the time of surgery was 58.2 years (range 40-73). The mean BMI of the patient sample was 30.75 kg/m^2^ at time of surgery. According to Cleveland Clinic BMI ranges, which indicate weight types: one patient (5%) was normal (BMI <25), 9 patients (45%) were overweight (BMI 25-29.9), 5 patients (25%) classified with class I obesity (BMI 30-34.9), 4 patients (20%) with class II obesity (BMI 35-39.9), and one patient (5%) classified as class III obesity (BMI >40) (Table 1).

**Table 1.** Patient Demographics and Operative Characteristics of the Study Cohort

Demographics	
Age, years (mean ± SD)	58.2 ± 10.02
Female, *n* (%)	15 (75%)
Male, *n* (%)	5 (25%)
BMI, kg/m^2^ (mean ± SD)	30.75 ± 4.94
BMI categories, *n* (%)	
Normal (<25)	1 (5%)
Overweight (25-29.9)	9 (45%)
Class I obesity (30-34.9)	5 (25%)
Class II obesity (35-39.9)	4 (20%)
Class III obesity (≥40)	1 (5%)
Comorbidities, n (%)	
Hypertension	16 (80%)
Diabetes mellitus	7 (35%)
Hyperlipidemia	3 (15%)
COPD	1 (5%)
Previous cancer	4 (20%)
Former smoker	2 (10%)
Previous abdominal surgery	15 (75%)
Follow-up, months (mean, range)	27.3 (10-48)
Types of prior abdominal surgeries, n (%)	
Cesarean section delivery	7 (35%)
Gastric bypass	6 (30%)
Gastrectomy	4 (20%)
Abdominal hysterectomy	3 (15%)
Abdominoplasty	2 (10%)
Abdominal trachelectomy	1 (5%)
Previous hernia repair	12 (60%)
Operative data (mean ± SD)	
Total operative time, min	306.3 ± 100.3
Reconstructive portion, min	114.9 ± 44.9
Cosmetic portion, min	166.7 ± 73.3
Resected tissue weight, kg	2.93 ± 2.76
Mesh used, *n* (%)	10 (50%)^a^

^a^Per-hernia = 10/23 (43.5%).

Regarding comorbidities, 16 patients (80%) had hypertension, 7 patients (35%) presented with diabetes mellitus, 3 patients (15%) with hyperlipidemia, one patient (5%) with COPD, 4 (20%) with previous cancers, 2 (10%) with a history of smoking, fifteen (75%) with previous abdominal surgeries (eg, prior cesarean section, abdominoplasty, abdominal wall reconstruction), and twelve patients (60%) had prior surgeries associated with their current hernias. Overall, mean total operative time was 306.3 minutes (range: 180-510 minutes) (Table 1).

Across 20 patients, 23 hernias were repaired (3 patients had 2 hernias). Hernia distribution consisted of 9 ventral/incisional (39.1%), 4 umbilical (17.4%), 3 inguinal (13.0%), 2 recurrent ventral (8.7%), 2 epigastric (8.7%), one complex ventral (4.3%), one parastomal (4.3%), and one recurrent inguinal (4.3%). Mesh reinforcement was used in 10/23 hernias (43.5%). For the patient with the parastomal hernia, the hernia was addressed at the time of stoma takedown with restoration of bowel continuity by the general surgery team; abdominoplasty was performed after fascial closure (Table 2).

**Table 2.** Hernia and Surgical Characteristics of Repair

Hernia type	*n* (%)	Surgical technique	Suture type^a^	Reinforcement method^b^
Ventral incisional	9 (39.1%)	Open: 9	Nonabsorbable (Polypropylene/Ethibond): 8 Long-acting absorbable (PDS): 1	Mesh: 2 Primary closure: 6 Closure w/retention of prior mesh: 1
Umbilical	4 (17.4%)	Open: 3 Laparoscopic: 1	Nonabsorbable: 4	Mesh: 2 Primary closure: 2
Inguinal	3 (13.0%)	Open: 3	Nonabsorbable: 3	Mesh: 2 Primary closure: 1
Recurrent ventral	2 (8.7%)	Open: 2	Nonabsorbable: 2	Primary closure: 1 Closure w/retention of prior mesh: 1
Epigastric	2 (8.7%)	Open: 2	Nonabsorbable: 2	Mesh: 1 Primary closure: 1
Complex ventral	1 (4.3%)	Open: 1	Nonabsorbable: 1	Primary closure: 1
Parastomal	1 (4.3%)	Open: 1	Long-acting absorbable: 1	Primary closure: 1
Recurrent inguinal	1 (4.3%)	Open: 1	Nonabsorbable: 1	Mesh: 1
Total hernias repaired	23 (100%)	Open: 22 (95.7%) Laparoscopic: 1 (4.3%)	Nonabsorbable: 21 (82.6%) Absorbable: 2 (17.4%)	Mesh: 10 (43.5%) Primary: 11 (47.8%) Closure w/retention of prior mesh: 2 (8.7%)

^a^Ethicon, a Johnson & Johnson MedTech Company.

^b^PROLENE Polypropylene Mesh, a Johnson & Johnson MedTech Company.

Reinforcement with mesh was based on defect size. Synthetic polypropylene mesh was preferentially used in clean cases with BMI <35. Acellular dermal matrix (ADM) was also considered and reserved for patients with contaminated surgical sites or high-risk patients that could have imposed complications with meshes, however, no patients in this series required the use of ADM. Average hernia repair mesh size was 69.3 cm^2^, (range: 6-500 cm^2^).

In several patients, the extent of the ventral hernia and associated attenuation of the umbilical stalk required intraoperative excision of the native umbilicus, which was a decision collaboratively decided upon by the multidisciplinary surgical teams. In 3 (15%) such cases, a neoumbilicoplasty was performed using a transcutaneous purse-string technique with dermal fixation to the rectus fascia. This approach was chosen for its ability to create a natural-appearing depression with minimal abdominal dissection and tension on the central flap. The purse-string method is commonly used for such closures to allow for even distribution of tension around the defect and preserving vascular supply, minimizing localized ischemia or bleeding caused by the suture.^13^ No umbilical necrosis was observed in our patient sample.

All patients were managed as outpatient procedures with same-day discharge and completed 30-day follow-up. Longer-term surveillance for hernia recurrence was available in 11/20 patients, with a mean follow-up of 27.3 months (range 10-48 months). The overall minor complication rate was 15% (3/20 patients), with no major complications observed. Importantly, no patients presented with postoperative hematoma formation, surgical site infections, or skin necrosis. Two patients (10%) developed postoperative seromas. Both seromas were managed conservatively with serial aspirations and resolved without requiring surgical intervention. One patient (5%) experienced chronic drainage. Approximately 5 months after the initial operation, the patient presented with 2 draining sinus tracts along the abdominal incision. Evaluation revealed partial wound dehiscence with formation of a larger contiguous wound. The drainage was presumed to be secondary to retained foreign bodies, likely suture material, impairing wound healing and epithelialization. The patient underwent bedside removal of the foreign material and intermediate closure of 2 abdominal wounds measuring 4.0 and 2.5 cm, respectively. The wounds subsequently healed without further intervention, and no hernia recurrence or mesh-related complications were observed. No hernia recurrences or reoperations were identified at last follow-up. Notably, none of the patients required revision of either hernia repair or abdominoplasty.

## DISCUSSION

This retrospective study evaluated outcomes following combined hernia repair and abdominoplasty procedures, demonstrating favorable outcomes across a cohort of twenty patients. Our findings suggest that when patient selection and perioperative optimization are carefully managed, the integration of reconstructive and aesthetic goals can safely be achieved with acceptable rates of surgical morbidity and perioperative complications.

Among the patient sample, postoperative complications were infrequent. The observed seroma rate of 10% aligns closely with existing literature citing seroma formation rates between 8% and 12.5% following abdominoplasty alone, suggesting that the addition of concurrent hernia repair does not significantly increase this risk.^14^ Such occurrences, although rare, are recognized as sequelae following combined hernia repair and abdominoplasty procedures, particularly in cases involving foreign body reaction or local tissue ischemia.^15,16^ This finding is supported by recent work from Phan et al, who demonstrated similar complication profiles in combined procedures.^15^ Overall, this low complication rate supports the growing body of evidence that, in selected patients, concurrent hernia repair and abdominoplasty may be performed without a substantial increase in postoperative risk.^10,17^

Recent literature has increasingly supported the safety of combined procedures. A 2024 narrative review by Chua et al examined techniques for preserving umbilical blood supply during combined ventral hernia repair and abdominoplasty, finding that meticulous surgical technique could minimize umbilical necrosis risk.^18^ In addition, the review reported complications being few, minor and primarily compounded by patient-specific risk factors as opposed to combined nature of the procedures. Our study findings align with this evolving literature, as we observed no umbilical necrosis in our patient cohort despite performing concurrent hernia repairs and abdominoplasty in patients with diverse hernia types and anatomical complexity, given their comorbidities. While the combination of traditional procedures was historically considered unfeasible due to umbilical blood supply compromise and increased necrosis risk, technical modifications have made simultaneous repair both feasible and safe. Our 0% umbilical necrosis rate and 15% minor complication rate support this shift toward multidisciplinary surgical approaches when appropriate surgical expertise and careful patient selection are employed.

The economic and patient-centered benefits of concurrent surgeries are substantial. Patients undergo a single anesthetic exposure, experience one recovery period, and typically return to work sooner than with staged procedures. The consolidated recovery period reduces overall time away from work and daily activities, providing both economic and quality-of-life benefits for patients. Likewise, concurrent procedures are beneficial for health centers by increasing patient-procedure allocation, improving patient throughout and a better utilization of hospital, physician and medical staff resources when taken together.^19^

A critical factor in achieving these outcomes appears to be rigorous preoperative patient optimization.^20^ Consistent with the broader surgical literature,^6^ our findings underscore the critical importance of achieving a stable weight prior to surgery, ideally with maximum weight loss sustained for at least 6 months. In patients with a history of bariatric procedures, ensuring adequate correction of micronutrient deficiencies remains essential to minimizing wound healing complications. These suggestions align with prior evidence from Benotti et al,^21^ who demonstrated that preoperative weight loss and nutritional optimization are associated with reduced postoperative morbidity.

Furthermore, the sequence of repair deserves special attention. When midline defects were present, we performed mesh placement before rectus plication, creating a reinforced repair without mesh-fascial edge tension. This “sandwich” technique (mesh-fascia-plication) may contribute to our zero-recurrence rate.

Our use of permanent polypropylene sutures for fascial closure, while providing durable strength, was associated with our only case of chronic drainage. Recent evidence from Haskins et al^22^ on standardizing wound event reporting emphasizes the importance of tracking such complications to improve surgical techniques. Permanent nonabsorbable polypropylene sutures were selected based on the surgeon's preference for fascial closures. The literature on suture materials for abdominal wall and rectus diastasis repair is not consistent, with studies exemplifying that both slowly absorbable (such as PDO) and nonabsorbable sutures (such as polypropylene) offer durable outcomes with low rates of infection.^23^ Multiple studies have supported the use of nonabsorbable sutures as a safe and durable suture material for abdominal closure as we have found in our practice as well.^23-26^

Recurrence and delayed wound events typically manifest within the first 12 to 24 months after ventral hernia repair, with late recurrences possible thereafter. Surgical site occurrences (seroma, superficial infection, minor dehiscence) are most commonly observed within 30 days, though persistent seroma or late drainage can occur in subsequent months. However, in our mean follow-up of 27.3 months, there were no recurrences, supporting durable repair with our technique. Nevertheless, more research must be done to ensure the safety of integrating both procedures that could mutually benefit both providers and patients seeking better health outcomes.

Further studies are warranted to provide an indication of whether a decrease in abdominal girth may lessen recurrent hernia formation and other commonly associated complications. Our findings indicate that this combined procedure has no significant impact on major complication rates, while the 10% seroma formation rate aligns with expected outcomes for abdominoplasty alone. Additionally, patients with a BMI exceeding 40 kg/m^2^ are known to carry a higher risk for perioperative morbidity in both hernia repair and body contouring surgeries and thus require individualized risk stratification and counseling.^26^ The increasing availability of GLP-1 receptor agonists offers new opportunities for preoperative optimization. Patients achieving substantial weight loss with these medications before surgery may have improved outcomes, though specific data on this population is still emerging.

This study had some limitations that warrant consideration. First, as a retrospective review conducted at a single academic center, our limited sample size of 20 patient procedures restricted the statistical power of our data analysis. While our complication rates of tissue necrosis, hematoma and seroma formation, hernia recurrence rate and several others aligned with current literature, larger multicenter studies would be needed to confirm these findings and establish a definitive safety profile for combined hernia repair and abdominoplasty procedures. Secondly, incomplete documentation of operative notes of 3 patients did limit our ability to analyze the time breakdowns between the aesthetic (abdominoplasty) and reconstructive (hernia repair) portions of this concurrent procedure. Nevertheless, the other time datapoints that were available were fairly consistent.

## CONCLUSIONS

This retrospective review demonstrates that combining hernia repair and abdominoplasty can be performed safely and reliably in appropriately selected patients. In our cohort of 20 patients, the overall minor complication rate was 15%, including a 10% seroma rate consistent with reported rates in the literature. One case (5%) of chronic drainage managed conservatively, and no cases of postoperative umbilical necrosis, hematoma or hernia recurrence were observed in the average 27.3 month follow up. Our results indicate that it is safe to perform abdominoplasty and repair abdominal hernias simultaneously. The absence of hernia recurrence and major complication in this series, despite the heterogeneity of hernia types and patient comorbidities, highlights the feasibility of this combined approach when performed by experienced multidisciplinary teams with careful patient selection criteria and meticulous surgical technique.
